# Trained Immunity: An Overview and the Impact on COVID-19

**DOI:** 10.3389/fimmu.2022.837524

**Published:** 2022-02-17

**Authors:** Justin M. Brueggeman, Juan Zhao, Madison Schank, Zhi Q. Yao, Jonathan P. Moorman

**Affiliations:** ^1^ Center of Excellence in Inflammation, Infectious Disease and Immunity, James H. Quillen College of Medicine, East Tennessee State University, Johnson City, TN, United States; ^2^ Division of Infectious, Inflammatory and Immunologic Diseases, Department of Internal Medicine, Quillen College of Medicine, East Tennessee State University (ETSU), Johnson City, TN, United States; ^3^ Department of Biochemistry and Cellular and Molecular Biology, University of Tennessee, Knoxville, TN, United States; ^4^ Hepatitis (HCV/HBV/HIV) Program, James H. Quillen VA Medical Center, Department of Veterans Affairs, Johnson City, TN, United States

**Keywords:** trained immunity, BCG, B-glucan, monocytes, natural killer (NK), COVID-19

## Abstract

Effectively treating infectious diseases often requires a multi-step approach to target different components involved in disease pathogenesis. Similarly, the COVID-19 pandemic has become a global health crisis that requires a comprehensive understanding of Severe Acute Respiratory Syndrome Corona Virus 2 (SARS-CoV-2) infection to develop effective therapeutics. One potential strategy to instill greater immune protection against COVID-19 is boosting the innate immune system. This boosting, termed trained immunity, employs immune system modulators to train innate immune cells to produce an enhanced, non-specific immune response upon reactivation following exposure to pathogens, a process that has been studied in the context of *in vitro* and *in vivo* clinical studies prior to the COVID-19 pandemic. Evaluation of the underlying pathways that are essential to inducing protective trained immunity will provide insight into identifying potential therapeutic targets that may alleviate the COVID-19 crisis. Here we review multiple immune training agents, including Bacillus Calmette-Guérin (BCG), β-glucan, and lipopolysaccharide (LPS), and the two most popular cell types involved in trained immunity, monocytes and natural killer (NK) cells, and compare the signaling pathways involved in innate immunity. Additionally, we discuss COVID-19 trained immunity clinical trials, emphasizing the potential of trained immunity to fight SARS-CoV-2 infection. Understanding the mechanisms by which training agents activate innate immune cells to reprogram immune responses may prove beneficial in developing preventive and therapeutic targets against COVID-19.

## Introduction

Severe Acute Respiratory Syndrome Corona Virus 2 (SARS-CoV-2), the most recent coronavirus to emerge, has led to the COVID-19 pandemic. As of December 5, 2021, SARS-CoV-2 has infected over 265 million individuals and resulted in over 5.2 million deaths globally (1). Currently, the available mRNA vaccines have been shown to induce and maintain an immune response for at least six months *via* the production of antibodies against SARS-CoV-2, contributing to reduced viral spread, hospitalization, and mortality (2); however, these vaccines cannot completely control SARS-CoV-2 infection. Moreover, new SARS-CoV-2 variants, such as the delta variant (B 1.617. 2) and the omicron (B. 1. 1. 529), have recently emerged and caused breakthrough infections despite prior vaccinations (3). Many severe cases of COVID-19 progress into acute respiratory distress syndrome (ARDS), mediated by the alteration of the innate immune system that leads to an overproduction of pro-inflammatory cytokines (cytokine storm) (4). Thus, a thorough understanding of innate immune activation during SARS-CoV-2 infection is necessary to develop additional prophylaxis and therapeutics.

There is evidence that innate immune cells are significantly altered during SARS-CoV-2 infection, with severe COVID-19 patients displaying lower levels of total monocytes (5) and a decreased number and activity of natural killer (NK) cells (6). A potential solution for treating coronavirus infections is training of the host’s innate immune system, termed trained immunity, which elicits immune protective effects in several cell types by activating specific signaling pathways. The clear involvement of the innate immune system in COVID-19 immunologic dysregulation necessitates an in-depth characterization of innate immunological mechanisms. Additionally, trained immunity may benefit elderly populations which have a general decreased immune response to vaccines (7). The field of trained immunity has demonstrated many advances in boosting the innate immune system against both bacterial and viral infections, and these may be applied to preventing and/or treating SARS-CoV-2 infection.

To evaluate trained immunity in the era of COVID-19, this review focuses on an overview of trained immunity mechanisms and their impact on COVID-19. Specifically, we describe both individual immune training agents and different innate immune cell types to distinguish specific pathways through which trained immunity can be induced. Furthermore, we relate these pathways to prospective trained immunity COVID-19 therapeutics using clinical trial data. Finally, we summarize the possible implications and future directions of trained immunity in COVID-19. A thorough understanding of these pathways and the subsequent trained immune responses is critical to evaluate how trained innate immunity can be exploited to SARS-CoV-2 infection.

## Trained Innate Immunity: A Method to Induce Heterologous Immunological Effects

Host immune responses can be distinguished as innate, which protects in a quick and nonspecific manner, and adaptive, which is slower to act but can induce specific and robust immunological responses *via* interactions between antigen presenting cells (APCs), such as dendritic cells (DCs) and B cells, and T cells. Although innate immunity is generally considered to be non-specific against invading pathogens, there is evidence that infections and inflammation can induce natural memory in innate immune cells (8). For example, natural killer (NK) cells can undergo expansion, similar to clonal expansion observed in adaptive immune cells, driven by pro-inflammatory cytokines such as IL-12 and IL-18 (9). Innate lymphoid cells (ILCs), which include cytotoxic ILCs (NK cells) and non-cytotoxic ILCs (ILC1, ILC2, and ILC3), are innate immune cells that can confer immunological memory. These ILCs are defined as cells that express cytokine receptors and are capable of transcriptional modifications to regulate tissue damage/repair and induce inflammation. However, unlike T or B lymphocytes, ILCs cannot undergo semantical rearrangement to generate antigen receptors required to confer antigen specificity (10). The presence of ILCs, which provide transcriptionally regulated immunological memory, suggests that the innate immune system is more complex than previously considered, warranting detailed investigations of how the innate immune system may be trained.

Given the recent challenges to the concept that innate immunity is naïve and cannot possess trained capabilities, the field of trained immunity has given rise to novel approaches of mediating robust immune responses. Priming induces alterations to the immune status and functional state of cells, which do not return to basal levels prior to secondary stimulation, allowing for an additive effect of stimuli. Alternately, trained immunity is defined as the capacity of the innate immune system to maintain immunological memory that can protect against secondary challenge following returning to the resting state *via* a persistent epigenetic signature (11, 12). Due to the identification of numerous training agents able to induce trained immunity, a trained immunity database (TIDB) has been assembled (13). Trained immunity has been utilized to epigenetically program several innate immune cells (monocytes, NKs, myeloid cells, neutrophils, and non-cytotoxic ILCs) to elicit a stronger immune response upon subsequent infection (14). Harnessing the capabilities of trained immunity for COVID-19 first requires an understanding of the pathways responsible for mediating this heterologous immunity and which training agent(s) induce these immunological modifications.

## Immune Training Agents Employ Multiple Pathways to Induce Trained Immunity

**Figure 1:**
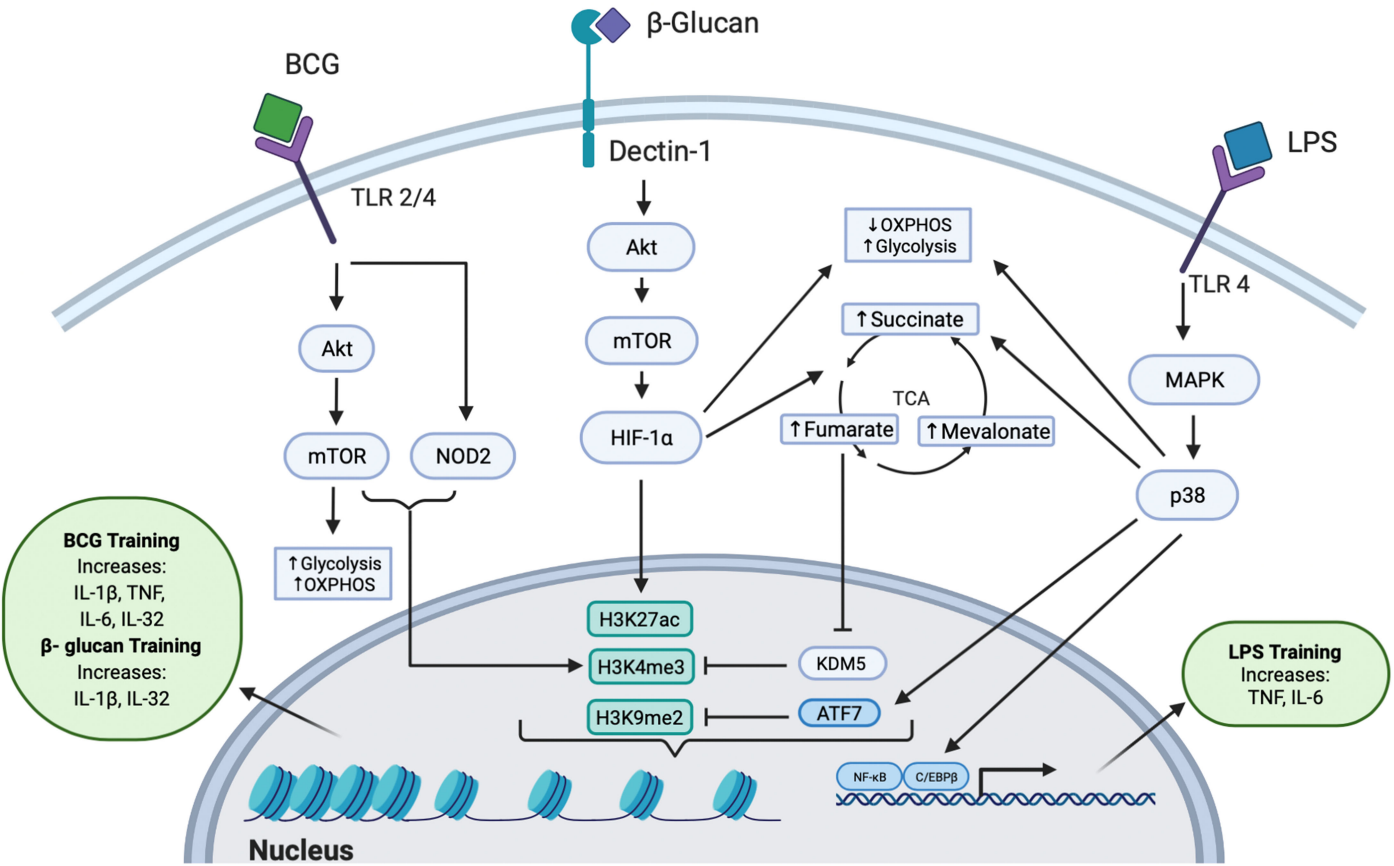
Intracellular Pathways Responsible for Trained Immunity. All three training agents train innate immunity to induce epigenetic modifications through multiple pathways. BCG binds to TLR 2 or TLR 4 and signals *via* the Akt/mTOR and NOD2 pathways, leading to downstream epigenetic modifications at H3K4me3. Activation of the Akt/mTOR pathway leads to upregulation of OXPHOS and glycolysis. The epigenetic change of H3K4me3 induced an increased production of IL-1β, TNF, IL-6, and IL-32 cytokines. β-glucan binds to the dectin-1 receptor to mediate downstream signaling through the Akt/mTOR/HIF-1α pathway. This signaling leads to decreased OXPHOS, increased glycolysis, and increased production of fumarate, succinate, and mevalonate in the TCA cycle. Fumarate further inhibits the KDM5 histone demethylase which acts at H3K4me3. β-glucan induced trained immunity was also shown to induce dynamic epigenetic changes at H3K27ac. The epigenetic changes further lead to increased IL-1β and IL-32 secretion. LPS can induce trained immunity by binding to TLR4 and signaling through the MAPK/p38 pathway, resulting in reduced OXPHOS, increased glycolysis, increased levels of succinate, and activation of transcription factors C/EBPβ and NF-κB. Additionally, LPS induces the phosphorylation and release of the inhibitory transcription factor ATF7, which inhibits the repressive histone mark H3K9me2. Collectively, these epigenetic changes upregulate the production of TNF and IL-6.

Trained immunity has been explored as a potential therapeutic against SARS-CoV-2 infection since the beginning of the COVID-19 pandemic. Several immune training agents, such as bacillus Calmette-Guérin (BCG), β-glucan, and lipopolysaccharide (LPS), have been used to stimulate innate immune cells into a protective state against subsequent challenges (**Figure 1**). Arguably the most notable training agent involved in inducing trained immunity is the live attenuated vaccine, BCG, intended to immunize against *M. tuberculosis* (15, 16). The BCG vaccine has been hypothesized to provide heterologous immunity against SARS-CoV-2 by epigenetically reprogramming monocytes, conferring an enhanced immune response upon secondary challenge (17). β-glucan, a polysaccharide isolated from *Candida albicans*, has also been found to possess immunogenic antiviral properties that could help reduce SARS-CoV-2 severity (18). LPS, which is commonly used to restimulate trained cells, can also act as a training agent by inducing persistent modifications in genes responsible for an increased response to secondary infection (19). Collectively, these data provide a foundation implicating these agents as inducers of trained immunity and propose their potential application in treating COVID-19.

### BCG

BCG can be used as a training agent *via* modulating intracellular signaling pathways by binding to both toll like receptor 2 (TLR2) and TLR4 to induce trained immunity (20). Subsequently, training monocytes with BCG activates the mammalian target of rapamycin (Akt/mTOR) pathway, leading to upregulation of glycolysis, oxidative phosphorylation (OXPHOS), and glutamine metabolism (21). In addition to the Akt/mTOR pathway, the nucleotide-binding oligomerization domain 2 (NOD2) pathway [activated *via* TLR2/4, which can induce IL-6, IL-1β, and tumor necrosis factor (TNF) (22, 23)], was found to be necessary for BCG-training of monocytes: blocking NOD2 by mutation or chemical inhibition resulted in BCG-trained monocytes that produced significantly lower amounts of TNF upon restimulation (24).

A study of severe combined immunodeficiency (SCID) mice treated with BCG demonstrated enhanced release of cytokines IL-1β and TNF in monocytes in a NOD2-dependent manner upon infection with *Candida albicans*, which was found to be epigenetically controlled by histone 3 lysine 4 trimethylation (H3K4me3), an open chromatin histone marker (24). Similarly, the Akt pathway was involved in BCG-trained neutrophils, inducing increased H3K4me3 patterns, specifically near promoter regions of genes encoding pro-inflammatory cytokines (*CXCL8* and *IL-1β*) and genes involved in glycolysis (*mTOR* and *PFKB*); this lasted at least three months post-vaccination (25). Epigenetic modifications at genes involved in signaling pathways such as *AKT1*, *MAPK*s, and *PI3K* (26) are responsible for inducing trained immunity and have been discovered within the epigenome of specific effector cells such as monocytes (27). This suggests that global epigenetic modifications also directly influence the “trained” functions of specific innate immune cells. Modulations in gene expression result in the increased production of cytokines involved in trained innate immunity. For example, IL-1β appears to be a critical cytokine induced by BCG-training: in BCG-trained monocytes, IL-1β levels increased after restimulation conferring non-specific protection against yellow fever viremia (26). The antimicrobial cytokine IL-32, which is known to be involved in viral defense and capable of modulating cellular metabolism (28), has also been demonstrated to play a role in BCG trained immunity, presumably by activating the NOD2 signaling pathway (29) (**Figure 1**).

Taken together, signaling pathways and epigenetic modifications induced by BCG-training are responsible for the enhanced innate immune response upon restimulation. Many of these signaling pathways are activated in different cell types within the innate immune system, suggesting that training with BCG may utilize conserved pathways. Since BCG vaccination has been demonstrated to possess therapeutic effects against respiratory infections, it has been hypothesized that BCG may offer heterologous immunity against SARS-CoV-2 infection (17, 30, 31). Therefore, ongoing clinical trials are investigating the relationship between BCG vaccination and protection against COVID-19. Currently, the data suggests that BCG-trained immune responses are present 3 months post BCG-treatment (24); thus, further research should be conducted to evaluate the cellular response of BCG-trained cells for longer times to determine the maximal duration of memory response and protection.

### β-Glucan

β-glucan is an immunomodulator that functions as a pathogen associated molecular pattern (PAMP) recognized by pattern recognition receptors (PRRs) expressed on innate immune cells. The immunomodulatory activity of β-glucan not only depends on its physical properties (for example, particle size, molecular weight, conformation, branching frequency, or solubility) but also on the cell type it acts upon, suggesting a highly diverse and targetable approach for β-glucan training (31). β-glucan signaling occurs in monocytes, macrophages, and DCs by the dectin-1 receptor, which activates an intracellular signaling cascade that drives the trained immune response (32–34). Upon β-glucan binding to dectin-1, the downstream Akt/mTOR/HIF1α pathway is activated, leading to subsequent metabolic modifications, including the downregulation of OXPHOS and upregulation of glycolysis (35), resulting in the increased production of tricarboxylic acid (TCA) cycle by-products, including succinate (36), fumarate (37), and mevalonate (a precursor for cholesterol synthesis) (38). In addition to binding to the dectin-1 receptor, β-glucan has been suggested to utilize other receptors to induce trained immunity (39).

Intracellular modifications of the TCA cycle driven through the Akt/mTOR/HIF1α pathway were found to play a key role in β-glucan trained immunity. Training with β-glucan was associated with increased levels of succinate, which has been suggested to have potential therapeutic benefits against sepsis-induced immunoparalysis (36). Fumarate, a metabolite involved in the TCA cycle, was found to increase cytokine production in a dose-dependent manner by inhibiting KDM5 histone demethylases, thereby leading to epigenetic changes (trimethylation, H3K4me3) at promoter regions of pro-inflammatory cytokine genes (37). Interestingly, KDM5B also regulates angiotensin-converting enzyme 2 (ACE2), the receptor involved in SARS-CoV-2 viral entry (40), providing a potential therapeutic target. Fumarate also stabilizes HIF1α by inhibiting hydroxylation, resulting in the induction of 2,688 dynamic H3K27ac changes, as revealed by chromatin immunoprecipitation followed by sequencing (ChIP-seq) (37). Monocyte training with mevalonate, indirectly synthesized from acetyl CoA, increased cytokine production upon restimulation. Importantly, inhibition of mTOR signaling or glycolysis impedes mevalonate training (38). Thus, β-glucan trained immunity is induced through distinct signaling pathways which drive metabolic and subsequent epigenetic modifications to maintain a sustained, non-specific response to invading pathogens (**Figure 1**).

Epigenetic changes induced by β-glucan in hematopoietic stem and progenitor cells (HSPCs) enable short-lived blood circulating monocytes to respond to secondary challenge, which leads to an increased level of IL-1β in trained monocytes, further inducing the production of IL-32 (41). Similarly, epigenetic reprograming of HSPCs by β-glucan drives trained immunity in an IL-1 dependent manner (42). Thus, β-glucan has a strong potential to be used as a training agent and already has prior applications as an oral supplement, which have been discussed in preventing SARS-CoV-2 infection (43). For example, since IL-1 is involved in COVID-19 dysregulated immune responses, recent studies have determined that blocking its receptor (IL-1R) can be used to treat severe COVID-19 infection (44). Since IL-1β production is greatly increased during COVID-19 (45), β-glucan trained immunity may enable the innate immune system to mount a stronger early immune response to prevent dysregulated cytokine production. This training of innate immune cells to increase production of key cytokines may ultimately improve the immune response upon SARS-CoV-2 infection.

### LPS

LPS, a microbial component comprising gram-negative bacterial membranes, binds to TLR4 and has primarily been used to restimulate cells after BCG or β-glucan training, but can also elicit trained immunity under certain conditions (46). Innate immune cells can be primed, resulting in transcriptional changes that provide an increased basal innate immune response (12). Prolonged priming with LPS results in reduced cytokine production and immunosuppression, inducing immune tolerance (47). These effects are due to reduced expression of TLR4, reduced TLR4/MyD88 interaction, and reduced activity of p38 (47). LPS immune tolerance was found to block the accumulation of histone marks at promotors or enhancers (H3K27ac and H3K4me1) of genes that are responsible for lipid metabolism and phagocytic pathways (48). LPS triggers transient silencing of inflammatory genes (associated with methylation at H3K9 and H3K27) as well as inducing antimicrobial effector genes (49). Training with LPS has been demonstrated to reduce inflammation and fibrosis in systemic sclerosis models by downregulating production of proinflammatory cytokines (IL-6, TNF, IL-1β) and instead upregulating production of the anti-inflammatory cytokine IL-10 (50, 51).

Conversely, lower doses of LPS treatment result in an increase of TNF and IL-6 upon restimulation (52), suggesting a role for LPS in trained immunity. LPS can induce trained immunity by activating innate immune cells like macrophages through chromatin and transcriptional modifications *via* signaling through the MAPK/p38 pathway **(** **Figure 1** **)** (11, 52). These include epigenetic modifications in myeloid cells, where treatment with LPS is associated with chromatin remodeling, such as an increased number of activating marks (53). Evidence suggests that these changes may be long lasting, since LPS stimulation of macrophages induced persistent methylation at H3K4 that corresponded to an enhanced response upon secondary stimulation (54). Additionally, LPS can induce trained immunity through regulation of transcription factors, like ATF7. LPS-treated macrophages were shown to induce phosphorylation and subsequent release of ATF7 *via* the p38 pathway, resulting in a decrease of repressive H3K9me2, which persists for long periods of time to increase resistance to subsequent reinfection (55). LPS has also been identified to induce epigenetic changes within hematopoietic stem cells (HSCs) that are dependent on the transcription factor C/EBPβ, which likely facilitates the long-lasting transcriptional changes shown to be induced by LPS (19). Similar to β-glucan, LPS treatment has been demonstrated to downregulate OXPHOS and upregulate glycolysis, resulting in increased levels of succinate (56), suggesting that LPS may induce trained immunity through multiple signaling pathways.

Finally, LPS has been demonstrated to indirectly decrease ACE2 expression *via* NF-κB-dependent upregulation of miR-200c-3p during H5N1 virus infection (57), suggesting that LPS may have additional preventative and therapeutic roles in COVID-19. Taken together, LPS training may provide beneficial effects to impair the ability of viral infectiousness and reduce severe inflammation in COVID-19.

### Alternative Immune Training Agents and Antagonists

In addition to BCG, β-glucan, and LPS, alternative immune training agents have been investigated, including aldosterone and oxidized low-density lipoprotein (oxLDL). Aldosterone-trained monocytes were characterized by enriched H3K4me3 at the promoter regions of genes involved in lipid synthesis (58). Similar to BCG treatment, oxLDL training upregulates both OXPHOS and glycolysis (59), which similarly results in increased TCA metabolites (60). Increased levels of proinflammatory cytokines IL-6 and TNF were mediated by the mTOR/HIF-1α pathway (61). These data suggest that oxLDL shares similar yet distinct pathways with BCG and β-glucan training, providing the opportunity to selectively train innate immunity by modifying metabolic pathways.

In contrast, several studies have highlighted that some reagents function as training antagonists, which can function to inhibit cell signaling or alter epigenetic modifications required to induce trained immunity. For example, BCG-incubated monocytes co-incubated with ARTA (a vitamin A metabolite) displayed reduced cytokine production, suggesting the role of ARTA as a trained immunity antagonist. This unique signature was determined to be caused by both increased expression of SUV39H2, a histone methyltransferase that induces the inhibitory histone 3 lysine 9 trimethylation (H3K9me3), and by downregulation of the stimulatory H3K4me3 (62). In addition to ARTA, some cytokines may also function as trained immunity antagonists. The anti-inflammatory cytokines IL-37 and IL-38 as well as the drug Hydroxychloroquine have demonstrated inhibitory effects against trained immunity. Both IL-37 and IL-38 inhibit β-glucan-mediated trained immunity by blocking mTOR signaling (63). IL-37 inhibits β-glucan trained immunity by reversing immunometabolic and histone modifications, thus suppressing production of pro-inflammatory cytokines (64). Likewise, hydroxychloroquine acts through the Akt/mTOR pathway to inhibit β-glucan trained immunity by preventing the production of TNF, IL-6, and IL-1β cytokines *via* blocking epigenetic modifications (65). Taken together, these trained immunity antagonists may be used with training reagents simultaneously to regulate the level of the immune response.

## Diversity of Cell Types That Can Develop Trained Immunity

In addition to evaluating the general intracellular mechanisms associated with trained immunity based on individual immune training agents, it is also important to identify the innate immune cell types capable of being trained. As listed in **Table 1**, immune training agents and antagonists have been demonstrated to induce or suppress trained immunity in different cell types with varying effects and durations. The innate immune system consists of a variety of different cells, of which monocytes and NK cells have demonstrated adaptive immune characteristics similar to T cells (71–73). Monocytes undergo epigenetic programing, modifying key metabolic pathways that enable an enhanced response to subsequent stimulation (74). Similarly, epigenetic reprograming of NK cells results in clonal expansion and cytokine secretion (11), which may also play a role in activating monocytes during their development (75). Understanding the cell-specific mechanisms through which trained immunity is induced is necessary to selectively target and modify host immunity to induce a stronger innate immune response upon SARS-CoV-2 exposure.

**Table 1:** Trained immunity agents and antagonists.

Modulator	Function	Training Effects	Cell Type	Duration	References
BCG	Training Agent	Increased glycolysis, oxidative phosphorylation and glutamine metabolism. Increased TNF and IL-6 production. Increased H3K4me3	Monocytes	3 months	(66)
		Increased ROS and phagocytosis, production of cytokines, epigenetic changes (H3K4me3)	Neutrophils	3 months	(25)
		Increased cytokine production (IL-1β, IL-6, and TNF)	NK cells	3 months	(16)
β-glucan	Training Agent	Increased TNF, IL-6, IL-1b, IL-32 cytokine production *via* metabolic pathways (itaconate, fumarate, glutaminolysis, mevalonate) which are driven by epigenetic changes and modulation of myeloid progenitor cells	Monocytes	3 months	(36–38, 41, 51, 67)
Aldosterone	Training Agent	Induces trained immunity *via* fatty acid synthesis, driven by H3K4me3 modifications	Monocytes	6 days	(58)
Catecholamines	Training Agent	Increased production of TNF and upregulation of glycolysis and oxidative phosphorylation	Monocytes	6 days	(68)
oxLDL	Training Agent	Increased cytokine production (IL-6, TNF) *via* mTOR, citric acid cycle metabolites, mitochondrial function, and oxidative phosphorylation	Monocytes	6 days	(60, 61, 69, 70)
LPS	Training Agent	Increased bacterial clearance due to induction of open chromatin regions within HSCs dependent on the transcription factor C/EBPβ	HSCs	3 months	(19)
IL-37	Training Antagonist	Inhibited beta-glucan induced trained immunity by blocking mTOR/AKT/HIF-1alpha signaling, resulting in blocked epigenetic modifications, metabolic changes, and cytokine production	Monocytes	N/A	(64)
IL-38	Training Antagonist	Inhibited beta-glucan induced trained immunity by blocking mTOR signaling and subsequent epigenetic changes	Monocytes	N/A	(63)
ATRA (Vitamin A)	Training Antagonist	Decreased cytokine production after BCG training by increasing expression of histone methyltransferase SUV39H2, inducing inhibitory mark at H3K9me3	Monocytes	N/A	(62)
LPS	Training Antagonist	Acts to instill “immune tolerance” characterized by blocking epigenetic changes	Macrophages	N/A	(48)

N/A, not applicable.

### Monocytes in Trained Immunity and COVID-19

Monocytes are critical innate immune cells that have a relatively short lifespan, circulating in the blood stream for 3 to 5 days (76). Despite their short lifespans, monocytes have been effective in presenting trained immune responses, which are driven by epigenetic histone modifications and have been shown to last at least 3 months (24), suggesting that monocyte reprograming may occur within the bone marrow and progenitor cells (11). Recent studies have classified trained immune responses as belonging to central trained immunity (occurring in bone marrow progenitor cells) and peripheral trained immunity (in blood monocytes and tissue macrophages). Monocyte reprogramming occurs at both levels, first by reprograming HSCs in the bone marrow followed by migration and potential differentiation into macrophages within the blood (74).

Epigenetic modifications after monocyte training drive subsequent changes in metabolic signatures. β-glucan trained monocytes exhibit increased glucose consumption and a shift from OXPHOS towards aerobic glycolysis (35), similar to what is observed in cancer metabolism (77). Epigenetic changes may be more dynamic than a few chromatin modifications, as training monocytes with β-glucan caused histone acetylation changes which resulted in approximately 8,000 dynamic regions (51). Taken together, these studies demonstrate that epigenetic modifications are responsible for sustained training effects in monocytes.

Monocytes have been shown to be involved in the innate immune response to COVID-19 (78). Several pro-inflammatory proteins, such as CXCL-10, IL-6, IL-10, IL-12, and TNF, have been identified to be upregulated in severe COVID-19 patients. Upregulation of these chemokine and cytokines were related to increased leptin, a hormone that signals through the NF-κB and STAT3 pathways (79). Similar to the effects of trained immunity, epigenetic changes such as open chromatin marks were present in monocytes from acute-COVID-19 patients and persisted six months after recovery from COVID-19 (80). Peripheral monocytes from COVID-19 patients exhibited mitochondrial dysfunction as evidenced by reduced glycolysis (81). Since trained immunity in monocytes is characterized by an upregulation of glycolysis (35), *in vivo* training of monocytes may help counteract this reduced glycolysis in COVID-19 patients. Identifying similar monocyte signatures during SARS-CoV-2 infection and innate immune training may help determine a role for trained immunity to prevent or treat COVID-19.

### NK Cells in Trained Immunity and COVID-19

Although NK cells are part of the innate immune system, they also share certain memory characteristics of adaptive immune cells (11, 82). NK cells can respond to host infections by recognizing cytokines IL-12 and IL-18 and producing interferon-gamma (IFN-γ) and TNF, as well as excreting perforin and granzymes which penetrate the membrane of an infected cell and induce apoptosis (83). Most innate immune cells are typically considered to be short lived; however, NK cells can live for longer than 6 months in both lymphoid and non-lymphoid organs (84). Similar to cytotoxic T cells, it has been shown that NK cells specific to a cytomegalovirus (CMV) virus receptor have a lifespan of several months (85). Memory NK cells are distinguished by their abilities of enhanced mitochondrial fitness, regulation, and OXPHOS capacity (86).

Although NK cells trained by BCG have been shown to upregulate the production of pro-inflammatory cytokines (IL-1β, IL-6, TNF) (16), the specific mechanism by which trained immunity is induced in NK cells is still poorly understood. The general mechanisms of NK memory and proliferation have been studied in murine models. Not surprisingly, many NK memory-like functions are driven by epigenetic modifications. In CMV infected mice, NK cells were able to mount adaptive immune functions which were subsequently identified to be a result of DNA hypermethylation patterns, similar to cytotoxic T cells (87). *In vitro* stimulation of NK cells with cytokines IL-12 and IL-18 followed by adoptive transfer into immune deficient mice led to memory-like NK responses that lasted for three months (88). Similar to trained monocytes which are characterized by metabolic reprogramming, recent studies of NK cell metabolism have shown that key metabolic energy pathways such as OXPHOS and glycolysis drive NK anti-viral responses. Moreover, NK cells shift from cytotoxic proliferation to a quiescent state upon resolution of inflammation, indicating that NK cells are highly dynamic which is beneficial for the trained immunity process (86). Metabolic analysis demonstrated that after exposure to a high concentration of IL-15, NK cells became activated *via* the mTOR pathway; inhibition of this pathway rendered NK cells incapable of proliferating and mounting cytotoxic responses (89). Finally, NK cells may play an indirect role in trained immunity by modulating monocyte function. It has been determined that bone-marrow-resident NK cells promote monocyte effector functions by secreting IFN-γ (75). Alternatively, innate immune crosstalk may also occur in the opposite direction, as macrophages can activate NK cells through secretion of IL-12 and IL-18 or direct ligand interactions (90, 91) Thus, the crosstalk among innate immune cells should be further investigated in trained immunity.

Since NK cells possess memory-like characteristics, training NK cells may allow for a more robust innate response against COVID-19. In hospitalized COVID-19 patients, overall NK cell counts were low; however, the level of adaptive NK cells was increased (92). Furthermore, in severe COVID-19 patients, NK cells were functionally impaired due to elevated IFN-α and TNF signaling (93), suggesting that dysregulation of NK cells is involved in pathogenesis during SARS-CoV-2 infection. Studying the underlying mechanisms by which trained immunity may enhance NK cell function could illustrate the potential application of training NK cells as a COVID-19 therapeutic approach.

## Clinical Studies of Trained Immunity in COVID-19

The evidence generated from experimental research regarding the effects of trained immunity has led to its application in translational clinical research. The immune response induced by BCG vaccination in the context of trained immunity has already been thoroughly reviewed (94, 95). Recent studies have evaluated these heterologous effects in relation to respiratory disease. BCG vaccination induced nonspecific effects in reducing infant mortality by 38%, with fewer cases of respiratory infection, fever, and sepsis (96, 97). A cohort of 58,021 children who were vaccinated with BCG showed 17-37% risk reduction for suspected acute lower respiratory tract infections (98). Current opinion suggests that the heterologous effects derived from BCG vaccination may achieve COVID-19 protection, and as such several ongoing clinical trials are investigating the protective effects of BCG in relation to COVID-19 (17, 30, 99–102). Several phase 2/3/4 clinical trials are ongoing in multiple countries to evaluate the efficacy of BCG vaccination as a preventative and therapeutic agent against COVID-19 among health care workers and elderly populations (**Table 2**).

**Table 2:** Clinical Trials related to trained immunity in COVID-19.

Intervention	Title	Phase	Enrollment (participants)	Location	NCT Number
BCG	Use of BCG Vaccine as a Preventive Measure for COVID-19 in Health Care Workers (ProBCG)	Phase 2	1000	Brazil	4659941
	Clinical Trial Evaluating the Effect of BCG Vaccination on the Incidence and Severity of SARS-CoV-2 Infections Among Healthcare Professionals During the COVID-19 Pandemic in Poland	Phase 3	1000	Poland	4648800
	Prevention, Efficacy and Safety of BCG Vaccine in COVID-19 Among Healthcare Workers	Phase 3	908	Mexico	4461379
	BCG to Reduce Absenteeism Among Health Care Workers During the COVID-19 Pandemic (EDCTP)	Phase 4	1050	Denmark	4641858
	COVID-19: BCG As Therapeutic Vaccine, Transmission Limitation, and Immunoglobulin Enhancement (BATTLE)	Phase 4	1000	Brazil	4369794
	Application of BCG Vaccine for Immune-prophylaxis Among Egyptian Healthcare Workers During the Pandemic of COVID-19	Phase 3	900	Egypt	4350931
	BCG Vaccine in Reducing Morbidity and Mortality in Elderly Individuals in COVID-19 Hotspots	Phase 3	2175	India	4475302
	Reducing COVID-19 Related Hospital Admission in Elderly by BCG Vaccination	Phase 4	2014	Netherlands	4417335
	BCG Vaccination to Protect Healthcare Workers Against COVID-19 (BRACE)	Phase 3	10078	Australia	4327206
	BCG Vaccination for Healthcare Workers in COVID-19 Pandemic	Phase 3	500	South Africa	4379336
	Reducing Health Care Workers Absenteeism in Covid-19 Pandemic Through BCG Vaccine (BCG-CORONA)	Phase 3	1500	Netherlands	4328441
	Using BCG Vaccine to Protect Health Care Workers in the COVID-19 Pandemic	Phase 3	1293	Denmark	4373291
	Using BCG to Protect Senior Citizens During the COVID-19 Pandemic	Phase 3	1900	Denmark	4542330
	Efficacy of BCG Vaccination in the Prevention of COVID19 Via the Strengthening of Innate Immunity in Health Care Workers (COVID-BCG)	Phase 3	1120	France	4384549
	Bacillus Calmette-guérin Vaccination to Prevent COVID-19 (ACTIVATEII)	Phase 4	301	Greece	4414267
	BCG Vaccine for Health Care Workers as Defense Against COVID 19 (BADAS)	Phase 4	1800	United States	4348370
beta-glucan (ABBC1)	Efficacy and Tolerability of ABBC1 in Volunteers Receiving the Influenza or Covid-19 Vaccine	N/A	90	Spain	4798677
IFN beta-1b + Ribavirin	IFN Beta-1b and Ribavirin for Covid-19	Phase 2	96	Hong Kong	4494399
IFN beta-1b + Remdesivir	IFN-beta 1b and Remdesivir for COVID19	Phase 2	100	Hong Kong	4647695
IFN alpha-2b + Rintaolimod	Rintatolimod and IFN Alpha-2b for the Treatment of COVID-19 in Cancer Patients	Phase 1/2	64	United States	4379518
IFN-γ	Study of the Use of Nasal IFN-γ in Patients for the Prevention of Acute Respiratory Viral Infections, Including COVID-19	Completed	630	Russia	5054114
IFN beta-1b	Double Therapy With IFN-beta 1b and Hydroxychloroquine	Completed	60	Hong Kong	4350281
IFN-alpha2b	Pegylated Interferon - α2b With SARS-CoV- 2 (COVID-19)	Phase 2	40	Mexico	4480138
pegylated IFN-lambda	Interferon Lambda for Immediate Antiviral Therapy at Diagnosis in COVID-19 (ILIAD)	Phase 2	240	Brazil and Canada	4354259
IFN-alpha2b	Inhaled Interferon α2b for the Treatment of Coronavirus Disease 19 (COVID-19) (IN2COVID)	Phase 1/2	168	Chile	4988217
IFN beta-1a	Human Intravenous Interferon Beta-Ia Safety and Preliminary Efficacy in Hospitalized Subjects With CoronavirUS (HIBISCUS)	Phase 2	140	United States	4860518
IFN-beta1b	Treatment of COVID-19 by Nebulization of Interferon Beta 1b Efficiency and Safety Study (COV-NI)	Phase 2	146	France	4469491
IFN-beta	Study to Assess Efficacy and Safety of Inhaled Interferon-β Therapy for COVID-19 (SPRINTER)	Phase 3	610	United Kingdom	4732949
IFN-beta (SNG001)	Trial of Inhaled Anti-viral (SNG001) for SARS-CoV-2 (COVID-19) Infection	Phase 2	820	United Kingdom	4385095

N/A, not applicable.

In addition to BCG, β-glucan has also been demonstrated to strengthen immune responses by targeting macrophages, DCs, and NK cells. A 2014 clinical study of 60 children orally treated with β-glucan showed successful stimulation of mucosal immunity in children with chronic respiratory problems (103, 104). Administration of β-glucan has been shown to correlate with improved respiratory symptoms (105, 106). For example, a study demonstrated that marathon runners who received β-glucan post-marathon reported fewer respiratory symptoms (107). Currently, one clinical trial is investigating ABBC1, a β-glucan dietary supplement, as a potential supplement for improving the efficacy of COVID-19 vaccination (**Table 2**).

IFN treatments have also demonstrated anti-viral effects and have been proven to be involved in trained immunity responses (108). One study determined that IFN-γ secreted by CD8 T cells can activate alveolar macrophages to induce memory characteristics (109). Type I IFNs have been previously used to treat SARS and MERS (110, 111), suggesting IFNs may be used as a potential COVID-19 therapy (112, 113). A recent clinical trial showed COVID-19 patients treated with IFN β-1a experienced faster recovery (114) and reduced mortality (115, 116). Ongoing clinical trials are using different forms of IFN, including IFN α-2b, IFN β-1a, and IFN β-1b, to investigate their role against SARS-CoV-2 infection (**Table 2**).

Most clinical trials for trained immunity seek to evaluate the efficacy of immune training by measuring protective immune responses and comparing the number of SARS-CoV-2-infected individuals between experimental and placebo groups. Trained immunity clinical trials can allow for the determination of different protective immune responses in COVID-19 vaccinated or infected individuals. Thus, clinical trials investigating heterologous protective immune responses may lead to discovery of alternative clinical applications of trained immunity.

## Future Directions and Conclusions: A Potential Approach to Fighting COVID-19

The field of trained innate immunity has demonstrated that innate immune responses are more complex and adaptive than previously thought. Additionally, the use of different training agents has illustrated a variety of specific intracellular pathways that can be targeted. The past two decades of trained immunity research have provided sufficient preliminary *in vitro* and *in vivo* data to merit the ongoing COVID-19 clinical trials.

**Figure 2:**
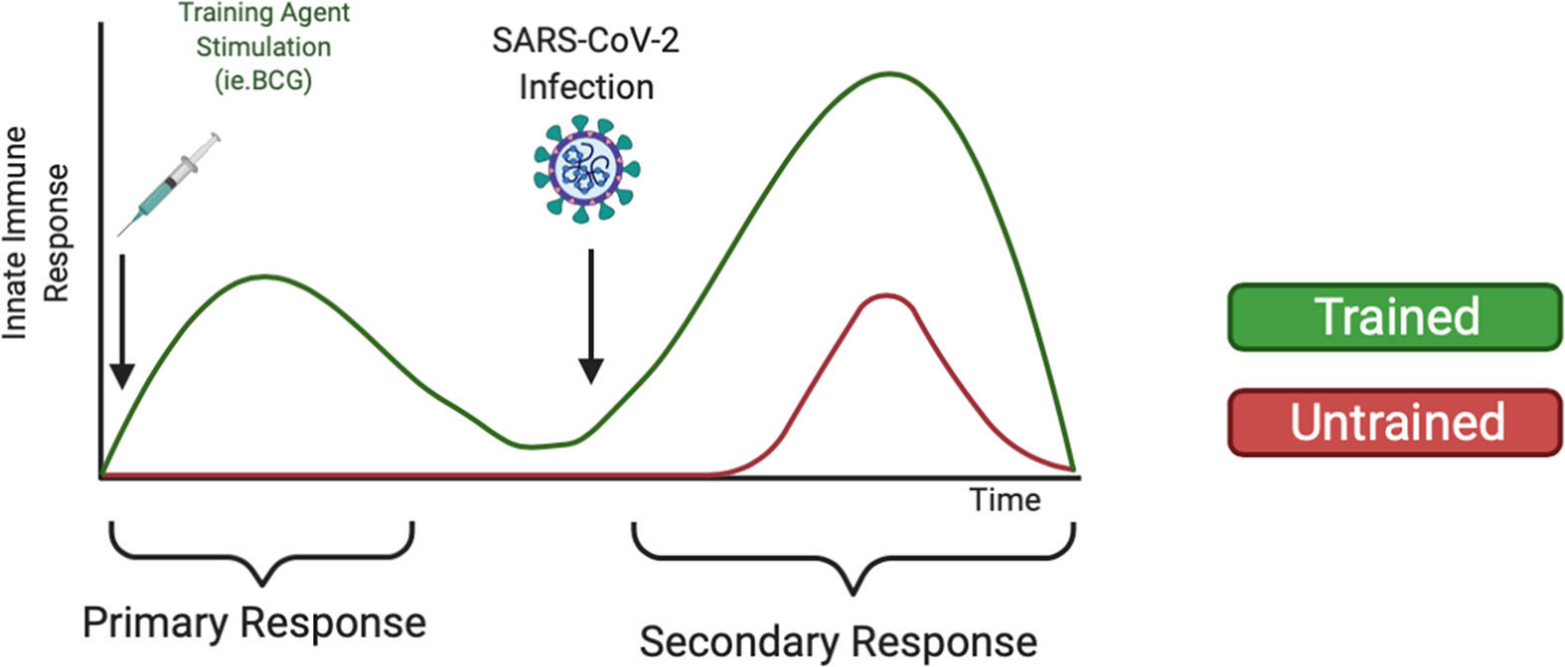
Heterologous Training of Innate Immunity Could Mediate Increased Secondary Response Upon COVID-19 infection. Trained immunity has been demonstrated to non-specifically train innate immune cells such as monocytes and NK cells against pathogens. Using training agents, like the BCG vaccine or β-glucan supplements, could strengthen the innate immune system to mount an increased innate immune response upon SARS-CoV-2 infection.

Although mRNA vaccines have proved effective against the alpha COVID-19 variant, there has been reduced efficacy against the delta and omicron COVID-19 variants (117). Breakthrough infections among vaccinated individuals are beginning to rise (118), likely due to the increased virulence and ability of SARS-CoV-2 variants to escape antibody neutralization (119, 120). These data warrant further research of alternative or supplemental methods to fight COVID-19 infection, such as training innate immunity (121), to prophylactically strengthen the innate immune system against SARS-CoV-2 infection. As shown in **Figure 2**, we expect individuals treated with training agents to mount a more robust innate immune response upon SARS-CoV-2 infection compared to untrained individuals. Additionally, we also expect that training innate immunity could be beneficial for COVID-19 vaccinated individuals by reducing breakthrough infections, disease severity, and mortality *via* enhanced immune responses. Furthermore, trained immunity may be beneficial in protecting the elderly against COVID-19 infection. Although aging is characterized by immunosenescence and a general dysregulation of both adaptive and innate immune systems, certain cell types remain preserved, including monocytes and NK cells (122, 123). Studies have highlighted the ability of BCG vaccination to induce the production of pro-inflammatory cytokines and reduce acute upper respiratory tract infections in elderly populations (124). Thus, utilizing trained immunity to prophylactically bolster preserved innate immune cells may improve elderly immune responses to COVID-19. However, immunosenescence may also affect epigenetic modifications such as histone methylation and acetylation (125). DNA hypermethylation at H3K9me3 and H3K27me3 has been associated with aging (126). Data from murine models has also shown that H3K9 acetylation decreased with age, suggesting that the presence of transcriptionally available chromatin decreases with age (127). Thus, further studies are needed to evaluate if epigenetic changes during immunosenescence alter trained immune responses. Data has also suggested changes in the presence and functionality of immune cells with increased age. Some cell types have been shown to have an age-related impairment, including neutrophils and DCs, while other cell types, such as NKs, have been shown to be preserved or even hyper-regulated with age (128, 129). Taken together, these data indicate the possibility that factors such as age may influence epigenetic reprogramming efficiency.

Recently, a considerable number of SARS-CoV-2-infected individuals have been shown to have “long COVID”, a condition marked by persistent symptoms lasting weeks to months after infection (130); however, the long-term impact of SARS-CoV-2 infection remains unclear. It is possible that persistent symptoms are a side effect of a dysregulated immune reaction during SARS-CoV-2 infection, which could be ameliorated with trained immunity approaches. Ongoing clinical trials assessing the immunogenicity of BCG, β-glucan, and IFNs against COVID-19 (100, 101) will hopefully provide promising evidence for COVID-19 prevention and therapeutics. To fully deploy a multi-faceted approach, it is important to explore all avenues of research, including the application of trained immunity, to investigate the underlying immunological processes that can be targeted for SARS-CoV-2 infection.

## Author Contributions

Conceptualization, JB and JM. Literature review, JB. Writing-original draft preparation, JB, JZ, and MS. Writing-review and editing, JB, JZ, MS, ZY, and JM. Supervisions: ZY and JM. All authors have read and agreed to the published version of the manuscript.

## Funding

This work was supported by American Diabetes Association award 7-20-COVID-149 (ZY) and Biomedical Laboratory Research and Development Merit Review Award (Department of Veterans Affairs, 5I01BX005428, JM).

## Author Disclaimer

The contents in this publication do not represent the views of the Department of Veterans Affairs or the United States Government.

## Conflict of Interest

The authors declare that the research was conducted in the absence of any commercial or financial relationships that could be construed as a potential conflict of interest.

## Publisher’s Note

All claims expressed in this article are solely those of the authors and do not necessarily represent those of their affiliated organizations, or those of the publisher, the editors and the reviewers. Any product that may be evaluated in this article, or claim that may be made by its manufacturer, is not guaranteed or endorsed by the publisher.
